# Single-molecule detection methods to study alpha-synuclein aggregation in postmortem Parkinson’s disease brains

**DOI:** 10.1016/j.crmeth.2026.101418

**Published:** 2026-04-23

**Authors:** Emre Fertan, John S.H. Danial, Stephen Neame, Jeff Y.L. Lam, Matthew W. Cotton, Melanie Burke, Zengjie Xia, Yunzhao Wu, Ben Powney, Yoichi Imaizumi, Annelies Quaegebeur, Georg Meisl, James Staddon, David Klenerman

**Affiliations:** 1 Yusuf Hamied Department of Chemistry, University of Cambridge, Cambridge CB2 1EW, UK; 2 UK Dementia Research Institute at University of Cambridge, Cambridge CB2 0XY, UK; 3 Eisai Ltd., EMEA Knowledge Centre, Mosquito Way, Hatfield AL10 9SN, UK; 4 Department of Clinical Neurosciences, University of Cambridge, Cambridge CB2 0XY, UK

**Keywords:** single-molecule detection, DNA-PAINT, SIMOA, soluble aggregate, diffusible aggregate, mouse model, Triton X-100, sarkosyl, extraction, data modeling

## Abstract

Nanoscopic aggregates of alpha-synuclein (ɑSyn) have been observed in Parkinson’s disease (PD). However, the processes that occur *in vivo* leading to the formation of these small aggregates are not well understood. We used ultra-sensitive single-molecule methods, including single molecule array (SIMOA), and super-resolution microscopy to quantify and characterize ɑSyn aggregates harvested from human brain samples, alongside a mouse model of synucleinopathy, using different tissue processing methods. While aggregate numbers did not differ between PD and control samples, larger aggregates were detected in PD brain samples. Moreover, different sub-populations of aggregates were obtained by different extraction methods, with diffusible and membrane-bound aggregates producing a more pronounced difference between disease and control samples. Our data suggest that ɑSyn aggregates slowly in the brain, leading to formation of larger aggregates in a sub-set of cells.

## Highlights

• Nanoscopic alpha-synuclein aggregates are involved in Parkinson’s disease pathology
• The small size and low abundance of these aggregates make them difficult to study
• Single-molecule detection methods can be used to study these aggregates
• Combined with data modeling, super-resolution imaging provides novel insights on PD

## Motivation

Involvement of nanoscopic alpha-synuclein aggregates in Parkinson’s disease has been demonstrated, but the small size, heterogeneity, and low abundance of these aggregates make them challenging to study. Moreover, different tissue processing methods harvest different sub-populations of these aggregates, leading to inconsistencies between findings. To address these issues, we used SIMOA and SiMPull, two single-molecule detection methods, to study alpha-synuclein aggregates in human postmortem brain samples and a mouse model of synucleinopathy. These tools enabled assessment of low picomolar concentrations of the aggregates, which are typically inaccessible with ELISA.

## Graphical abstract

**Figure:**
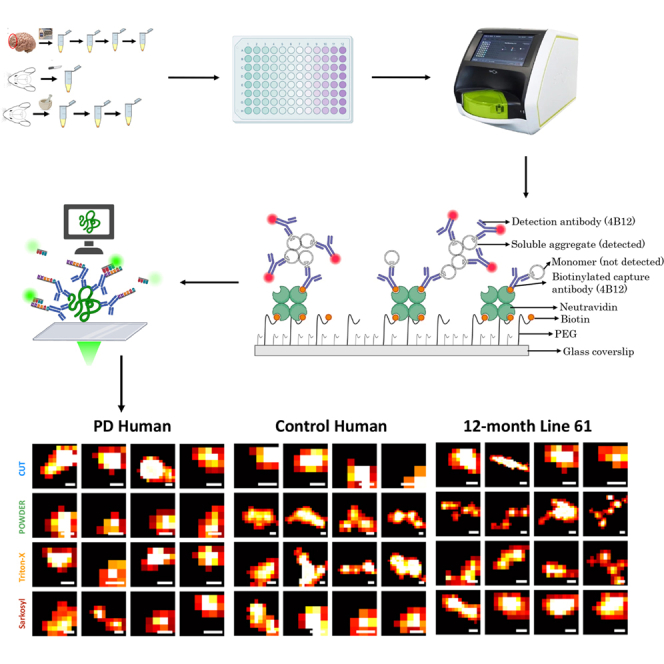

## Teaser

The nanoscopic aggregates of alpha-synuclein are believed to be involved in Parkinson’s disease progression. It is challenging to study these small aggregates with traditional microscopy techniques. Here, Fertan et al. use advanced single-molecule methods to characterize the small alpha-synuclein aggregates in human brain samples and mouse models.

## Introduction

Affecting ∼4% of the population over the age of 80, Parkinson’s disease (PD) is the most common neurodegenerative disorder leading to movement deficits.^1^ With motor symptoms of rigidity, bradykinesia, and resting tremor,^2^ accompanied by speech dysfunction,^3^ and in some cases cognitive impairment,^4^ PD significantly decreases quality of life^5^ and shortens the lifespan.^6^ While the exact cause(s) and neuropathological mechanisms of PD are still being studied, the loss of dopaminergic neurons in the substantia nigra pars compacta,^7^ as well as the aggregation and accumulation of alpha-synuclein (ɑSyn)^8^^,^^9^ have been well documented in PD brains.

As a synaptic protein, ɑSyn is required for neurotransmitter release by aiding the formation of the soluble N-ethylmaleimide-sensitive factor attachment protein receptor (SNARE) complex.^10^ However, in PD, ɑSyn forms large, insoluble aggregates called Lewy bodies,^8^ concentrating in regions where neuronal loss occurs, such as the substantia nigra.^11^ While the microscopic Lewy bodies consisting of fibrillar ɑSyn are the most commonly studied forms of ɑSyn aggregation and their atomic structures have been resolved using cryogenic electron microscopy (cryoEM), their role in the pathogenesis of PD has not been fully established. Meanwhile, some recent studies have shown that earlier forms of aggregation, namely nanoscopic aggregates—which are highly heterogeneous in size and shape^12^may be more toxic than Lewy bodies.^13^ Sometimes termed “oligomeric ɑSyn,” these smaller aggregates inhibit neurotransmitter vesicle docking and release.^14^^,^^15^ Our group has shown that these nanoscopic ɑSyn aggregates are present in the amygdala of the brains of patients with PD and, notably, also in brains from unaffected individuals, but the aggregates differ in size between PD and control cases, and aggregates under 200 nm in length are neurotoxic.^16^

While various studies have demonstrated the toxic properties of small aSyn aggregates, the exact species of these nanoscopic aggregates found in PD cases remains elusive, as it is challenging to characterize and study these aggregates. First, the small aSyn aggregates are highly heterogeneous, low in abundance, and their size is below the diffraction-limit of light, making them impossible to study and characterize with traditional microscopy techniques and bulk analysis methods, as they are smaller and less abundant than the resolution limit and sensitivity of these techniques. Single-molecule and super-resolution detection methods are therefore required to resolve individual aggregates so they can be quantified and characterized. Moreover, the methods used to process brain tissue and harvest aggregates often yield only a subset of the aggregates present in the tissue and may alter their properties and size distribution. This can bias observations toward species that are dominant for a particular extraction method. For instance, Hong et al.^17^ demonstrated that the method used to process postmortem Alzheimer’s disease brain samples impacts the type of beta-amyloid aggregates harvested. While gently soaking the samples gathers a small subset of aggregates, termed “diffusible,”^18^ these aggregates were highly toxic and capable of inducing long-term potentiation (LTP) deficits.^17^^,^^19^ Meanwhile, mechanical disruption of cellular integrity can harvest aggregates freely present in the cytosol but cannot solubilize membrane-bound aggregates. Since aSyn is known to interact with membranes and show oligomerization on lipid bilayers,^20^^,^^21^^,^^22^ detergents such as Triton X-100 (TrX) are needed to harvest aggregates bound to membranes.^23^ Lastly, harsher anionic detergents such as N-laurylsarcosine (sarkosyl) are known to destabilize fibrillar ɑSyn aggregates and release monomeric and aggregated species.^24^

In order to compare the nanoscopic aggregates harvested by different extraction methods and identify the “disease-relevant” species, we combined single-molecule and super-resolution detection methods with advanced data modeling. We first applied these methods to serially extracted (gentle soaking followed by homogenization, then TrX, and finally sarkosyl) human postmortem PD and control brain samples from the orbitofrontal cortex at Braak stages 4 and 5, which is prior to Lewy body pathology in this region (Table 1). We then studied the commonly-used Line 61 mouse model of PD to determine whether the mouse model mimics human disease in terms of disease-relevant small aggregates. Finally, we employed a mathematical model, which we have recently developed to interpret aggregate size distributions in terms of the underlying molecular mechanisms,^25^ to compare PD and control samples, along with the Line 61 mice.

**Table 1:** Demographic data for human orbitofrontal cortex tissue samples

Case	Diagnosis	Braak stage	Sex	Age
BB19.0032/NP19.108	PD	4	female	86
BB19.0010/NP19.023	PD	4	female	78
BB16.0014/NP16.162	PD	5	male	74
NP16.164/BB16.16	control	0	male	70
NP16.284/BB16.40	control	0	female	70
NP16.239/BB16.026	control	0	female	82

## Results

The small ɑSyn aggregates studied here are mostly below the diffraction-limit of light (∼200 nm) and thus cannot be resolved and characterized using traditional light microscopy techniques such as immunohistochemistry. Moreover, their high heterogeneity and low abundance make it difficult to separate disease-relevant species from larger (fibrillar) aggregates and monomers using bulk analysis methods such as ELISA. As such, single-molecule detection methods are required to quantify and morphologically characterize individual aggregates with high precision and compare them across different extraction methods. Here, we used an ultra-sensitive single-molecule array (SIMOA) assay developed by our group, which has been shown to quantify the amount of ɑSyn aggregates specifically (excluding monomers) in samples, with femtomolar sensitivity.^26^ Subsequently, the aggregates were characterized in terms of their size and shape using a combination of single-molecule pulldown (SiMPull) and DNA-PAINT imaging, previously developed and used by our group to characterize beta-amyloid^27^ and tau aggregates.^28^ In both assays, the same monoclonal antibody was used to capture and detect the targets; thus, aggregates are studied, excluding monomeric ɑSyn in the samples, as the single epitope is taken up by the capture antibody, making it impossible for the detection antibody to bind to the monomeric target. This was confirmed empirically by denaturation experiments and super-resolution imaging for SIMOA and SiMPull, respectively. Moreover, the total number of localizations (imaging strands bound to the aggregates) can be calculated in DNA-PAINT, positively correlating with the total target mass in the sample.^29^ The results of these experiments are presented below. To improve clarity the full results of statistical tests (including degrees of freedom, *p*-values, and confidence intervals [CIs]) are provided separately in the supplemental results file titled Data S1. Data are presented as Mean ± standard error of mean (SEM) in the graphs.

### ɑSyn aggregate concentration in human PD and control brain

**Figure 1:**
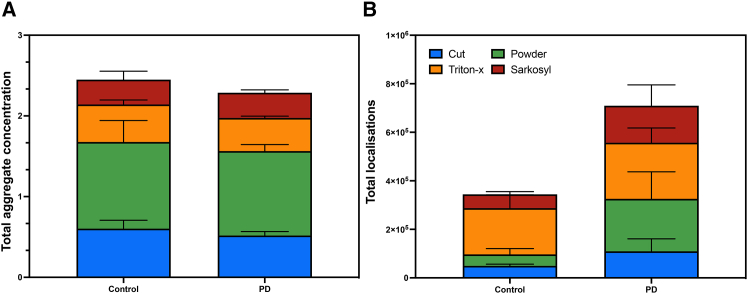
Quantification of alpha-synuclein aggregates in human brain samples (A) Total concentration of alpha-synuclein aggregates in postmortem human brain samples measured by SIMOA for soaking (blue), homogenizing (green), Triton X-100 (yellow), and sarkosyl (red) extractions. (B) Total number of localizations measured by DNA-PAINT super-resolution microscopy. Data presented as Mean + SEM.

**Figure 2:**
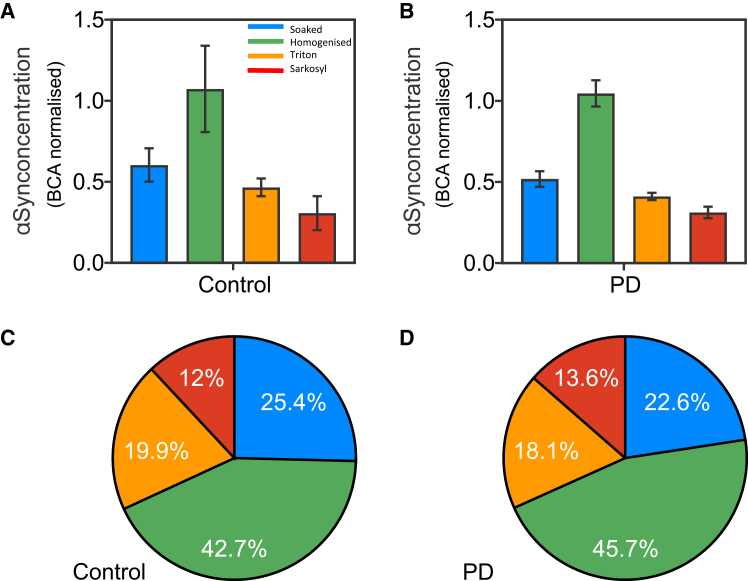
Extraction methods differ in terms of the quantity of aggregates they harvest from human brains Concentration of alpha-synuclein (ɑSyn) aggregates extracted by different methods from (A) control and (B) PD postmortem human brain samples using SIMOA (data presented as Mean±SEM), and the proportion of aggregates extracted by serial processing from (C) control and (D) PD samples.

We processed age- and sex-matched PD and control postmortem human orbitofrontal cortex samples with serial extractions and compared the quantity as well as the morphology of ɑSyn aggregates (see Graphical Abstract for sample images) using a purpose-built SIMOA aggregate assay and DNA-PAINT super-resolution microscopy, respectively. Strikingly, ɑSyn aggregate quantity did not differ between the PD and the control samples (Figure 1A), as the number of nanoscopic aggregates was not increased in the orbitofrontal cortex of PD brains. Meanwhile, there was a significant difference between aggregate quantities obtained using different extraction methods. The sarkosyl-soluble fraction contained the least number of aggregates for both disease and control samples, followed by the soaked and TrX-extracted fractions, and the homogenized samples contained the highest concentration of ɑSyn aggregates (Figures 1A and 2).

### PD brains contain larger aggregates than controls

While the concentration of nanoscopic ɑSyn aggregates did not differ between the PD and control orbitofrontal cortex samples, the morphology of these aggregates differed significantly. PD brains contained longer (mean of 80 vs. 65 nm) and larger (mean of 1360 vs. 770 nm^2^) aggregates with rounder (less fibrillar) shapes, regardless of extraction method, leading to a higher total mass of aggregated ɑSyn in PD brains (Figure 1B).

**Figure 3:**
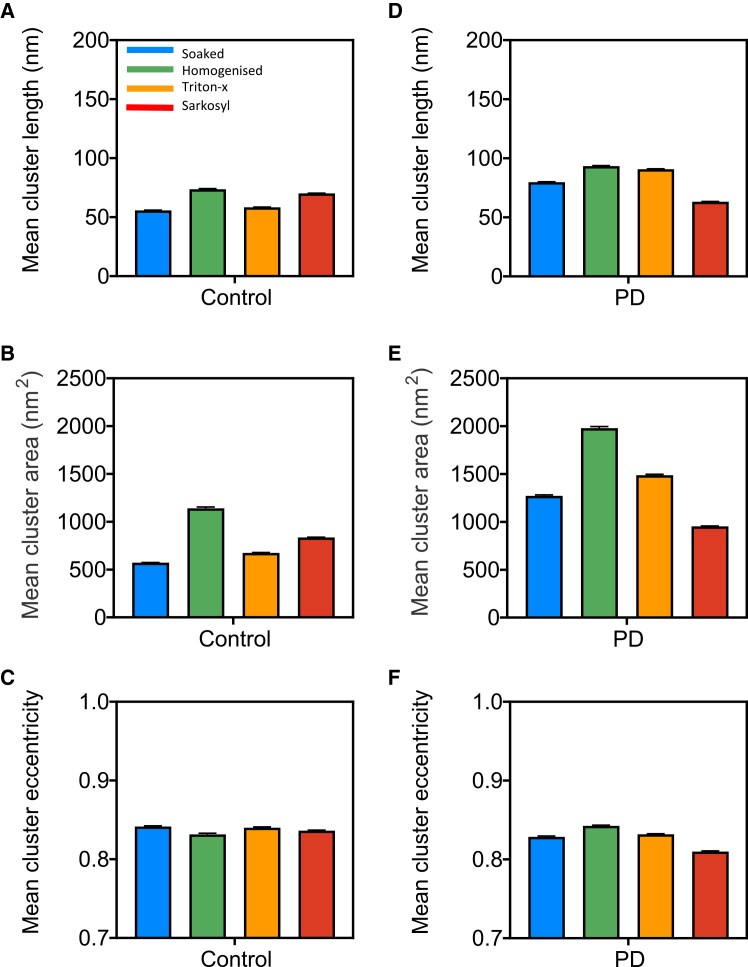
Extraction methods differ in terms of the morphology of aggregates they harvest from human brains Mean alpha-synuclein aggregate cluster length (nm), area (nm^2^), and eccentricity for aggregates harvested from control (A, B, C, respectively) and PD (D, E, F, respectively) postmortem human brain samples using gentle soaking (blue), homogenizing (green), Triton X-100 (yellow), and sarkosyl (red) extractions. Data presented as Mean±SEM.

Aggregate morphology showed further differences based on the extraction method. From the control brains, the longest (mean of 74 and 70 nm; Figure 3A), largest (mean of 1140 and 835 nm^2^; Figure 3B), and roundest (mean of 0.83 and 0.83; Figure 3C) aggregates were harvested by homogenizing and by sarkosyl extraction. However, the homogenized and TrX-extracted aggregates were longer (mean of 93 and 90 nm; Figure 3D) and larger (1980 and 1490 nm^2^; Figure 3E) in the PD brains, while the shortest (mean of 63 nm) and smallest (mean of 950 nm^2^) aggregates were in the sarkosyl-extracted samples. Unlike the control brains, the larger aggregates were more fibrillar in the PD brain, as the aggregates with the highest eccentricity were found in the homogenized fraction, followed by the TrX extraction, and the roundest aggregates were in the sarkosyl-soluble fraction (Figure 3F).

We have recently developed a mathematical model that allows us to interpret aggregate size distributions in terms of the underlying molecular mechanisms.^25^ Briefly, for aggregates well above the nucleation size, our model predicts a geometric decay of aggregate abundance with size. The rate of this decay is determined by the relative rates of aggregate formation and removal. When a cell is in a runaway aggregation, or “diseased” state, its rate of aggregate accumulation relative to removal is increased considerably, leading to a longer length distribution. In samples from PD cases, we expect some cells to still be “healthy,” producing shorter aggregates, and some cells to be “diseased,” producing larger aggregates. We quantified the balance of production and removal of aggregates in cells as the relative removal rate. This analysis of the size distribution yields three quantities: the relative removal rate in heathy (1) and diseased (2) cells, along with the fraction of aggregates originating from diseased cells (3).

**Figure 4:**
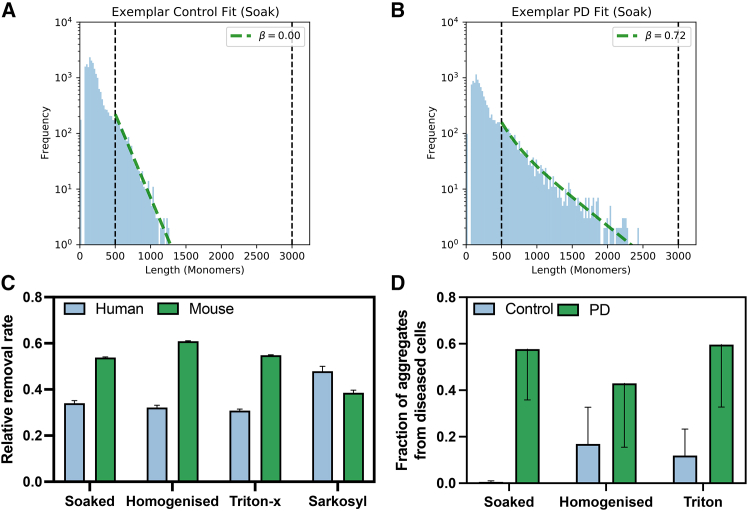
Mechanistic analysis of aggregate length distributions (A) Example histograms of the aggregate size distribution for control and (B) PD samples with soak extraction. The green dashed line shows the predicted distribution using the model and the mean parameters determined from Bayesian inference. (C) The difference in the mean β from the fitting procedure reflects the difference in the size of the disease subpopulation between the control and PD samples. This can also be seen directly from the longer tail in the PD dataset. The relative removal rate in diseased cells compared to healthy cells is also shown. For human samples from soaked, homogenized, and TrX extraction methods, the relative removal rate in diseased cells is decreased to 30% of the relative removal rate in healthy cells, consistent across the 3 extraction methods, indicating a considerable difference between the healthy and diseased states. For the mouse samples and the sarkosyl extraction for the human samples, this difference is less pronounced, with the relative removal rate in diseased cells being only about half of that in healthy cells. In samples from control brains, the majority of aggregates come from healthy cells, with the contribution from diseased cells being essentially undetectable in the soaked samples. (D) By contrast, in samples from individuals with PD, the majority of aggregates come from diseased cells. Data presented as Mean+/-SEM

In this experiment, we found that the relative removal rate in the healthy cell population is the same in both control and PD brains, implying that the aggregation behavior in healthy cells is the same in both PD and healthy aging. Similarly, the aggregates from diseased cells, which can also be detected at low levels in some control samples, behave similarly to aggregates from diseased cells in PD samples. Meanwhile, the main difference between PD and control brains is that the amount of aggregates from the diseased cells is significantly higher in PD, most likely due to the increased number of diseased cells in PD. Moreover, we found differences in the results depending on the extraction method: while the relative removal rate between the healthy and diseased states is comparable between the soaked, homogenized, and TrX-extracted samples, with the relative removal in diseased cells being only about 30% that of the relative removal in healthy cells, for sarkosyl the difference is only approximately 50%, making sarkosyl an outlier (Figure 4C). As such, we excluded the sarkosyl-extracted samples from the analysis of the fraction of aggregates that came from diseased cells. For the other three extraction methods, when we compared the fractions between the PD and control cases, the greatest difference was observed in the soaked samples, followed by TrX, while the homogenized samples showed the smallest difference (Figure 4D).

### ɑSyn aggregate concentration in Line 61 mouse brain

**Figure 5:**
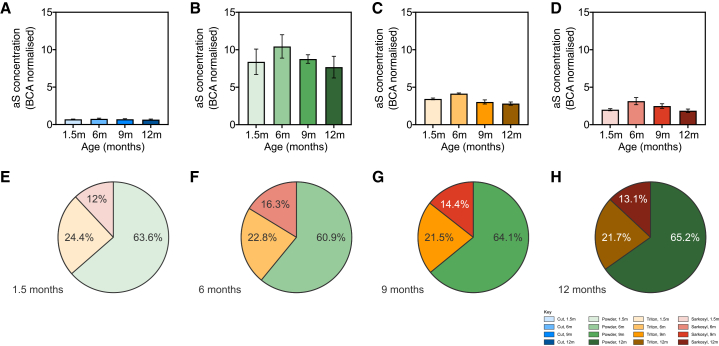
Extraction methods differ in terms of the quantity of aggregates they harvest from Line 61 mice Concentration of alpha-synuclein aggregates in Line 61 mouse brain measured by SIMOA for soaked (A), homogenized (B), Triton X-100 (C), and sarkosyl (D) extracted samples (data presented as Mean±SEM), and the proportion of aggregates extracted by serial processing at 1.5- (E), 6- (F), 9- (G), and 12-month (H) of age.

Following the human samples, we quantified the ɑSyn aggregate concentration in 1.5-, 6-, 9-, and 12-month-old Line 61 mouse brain samples extracted using the same methods, in order to compare aggregates produced in human brains with a mouse model expressing human ɑSyn at moderately high levels. While ɑSyn aggregate quantity did not differ between ages, the concentration of aggregates harvested by homogenizing, TrX, and sarkosyl extractions differed significantly (Figures 5A–5D). Similar to the human PD cases, the highest concentration of ɑSyn aggregates was found in the homogenized samples, followed by the TrX soluble extract, and the sarkosyl-soluble fraction (Figures 5E–5H). Meanwhile, the soaked samples, which were prepared using a brain from a different mouse, contained a smaller amount of aggregates compared to the other extraction methods.

**Figure 6:**
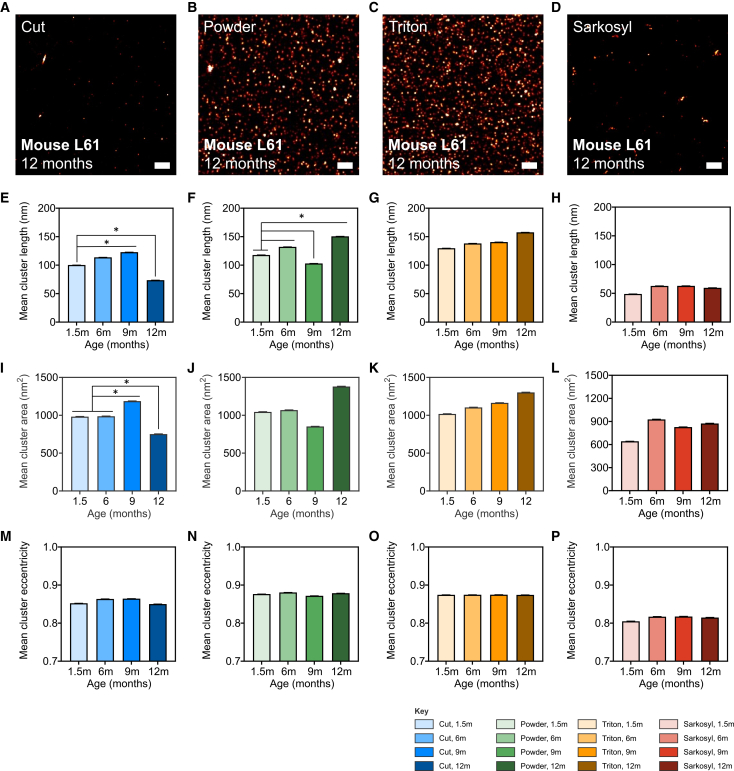
Extraction methods differ in terms of the morphology of aggregates they harvest from Line 61 mice Representative micrographs of super-resolved aggregates from 12-month-old Line 61 mouse brain processed by soaking (A), homogenizing (B), Triton X-100 (C), and sarkosyl (D) extraction (scale bars, 500 nm). Mean alpha-synuclein aggregate cluster length (nm), area (nm^2^), and eccentricity for aggregates harvested from Line 61 mouse brain by soaking (E, I, M, respectively), homogenizing (F, J, N, respectively), Triton X-100 (G, K, O, respectively), and sarkosyl (H, L, P, respectively) extraction. Data presented as Mean±SEM.

For the soaked samples, the average aggregate length was 100 nm at 1.5 months of age and increased to 114 and 123 nm at 6 and 9 months of age but decreased to an average of 74 nm at 12 months of age, suggesting a change in the morphology of soaked aggregates as the Line 61 mice age (Figures 6A and 6E). Aggregate area from the soaked samples followed a similar trend, with an increase at 9 months of age, followed by a decrease at 12 months (Figures 6A and 6I). Meanwhile, aggregate eccentricity did not change with age and had a value close to 1, indicating the presence of more fibrillar aggregates in all samples (Figure 6A & m). Within the serially extracted samples, the homogenized and TrX-soluble aggregates had an average length of 128 and 142 nm, respectively, and thus were longer than the sarkosyl-soluble aggregates, which had an average length of 59 nm. However, while the TrX-soluble aggregates grew longer as the mice aged, the homogenized aggregates varied in length and showed no clear trend with age. In contrast, while the sarkosyl-soluble aggregates grew longer after 1.5 months of age, they did not show any further significant age-related changes (Figures 6B–6D and 6F–6H). The area of the TrX-soluble aggregates grew larger with age, while homogenized and sarkosyl-soluble samples showed a fluctuating pattern (Figures 6B–6D and 6J–6L). However, eccentricity of the serially extracted samples differed by extraction method. While the homogenized and TrX-soluble aggregates were more fibrillar, the sarkosyl-soluble aggregates were more circular (Figures 6B–6D and 6N−6P).

### Comparing PD human and transgenic mouse aggregates

An essential question in the field of neurodegeneration and in the broader field of neuroscience is the validity of animal models. Since the morphology of pathological aggregates is related to their toxic properties,^16^^,^^30^ an important requirement of a useful mouse model for studying disease pathogenesis and testing novel therapeutics is likely to be that the aggregates formed are similar to those in humans. In order to evaluate the Line 61 mouse model of PD in terms of its aggregate validity, we once again employed our mathematical model. In all extraction methods except sarkosyl, the mouse data showed significantly less difference between the healthy and the diseased cell states than the human data, suggesting a decrease in the relative rate of removal of only 50%–60% (Figure 4C). In other words, while there is clear evidence for a healthy and a diseased cell population in both PD and control human brains, in the mouse, the distinction between two such populations is less clear. Furthermore, the length distributions observed in the mouse samples suggest that the relative removal rate in mouse cells corresponds to neither the healthy nor the diseased cell state in humans. For soaked and homogenized samples, the mouse rates lie between the healthy and diseased values for human samples. Meanwhile, for TrX, the relative removal rate in the mouse is always lower (Figure 4C).

## Discussion

In this study, we quantified and characterized nanoscopic ɑSyn aggregates from human postmortem PD and control orbitofrontal cortex samples, along with the commonly used Line 61 mouse model of PD. We processed the tissue using serial extractions with four commonly used methods to harvest different types of nanoscopic aggregates. Gentle soaking was used initially to harvest diffusible aggregates, followed by detergent-free homogenization to solubilize aggregates that are not diffusible but also not membrane-bound. Then the tissue samples were further treated with TrX to digest the cellular membrane and solubilize membrane-bound aggregates, followed by a sarkosyl extraction, which partially solubilizes fibrillar aggregates. As such, we were able to address five questions: (1) how do ɑSyn aggregates from postmortem PD brain samples compare to controls? (2) do different extraction methods harvest morphologically different types of aggregates? (3) does the quantity or morphology of ɑSyn aggregates change as Line 61 mice age? (4) how do ɑSyn aggregates from a mouse model compare to human samples? and (5) can the aggregate size distribution be fitted by chemical kinetics and what does this reveal about the aggregation mechanisms of ɑSyn *in vivo*?

The human postmortem tissue samples were selected from the orbitofrontal cortex (Table 1). Since this region has extensive dopaminergic input from the substantia nigra, executive dysfunction is one of the major symptoms of PD^31^ and orbitofrontal cortex volume and pathology is linked to deficits in reward processing and decision making.^32^^,^^33^ We used samples from Braak stage 4 and 5 brains, which is prior to Lewy body formation in this region, in order to characterize nanoscopic aggregates in a disease-relevant region before significant Lewy body pathology and neuronal loss.^34^

Remarkably, the aggregate numbers did not differ between PD and control brains, as the orbitofrontal cortex samples from non-PD brains also contained aggregates at comparable concentrations to PD brains. However, the aggregates in the PD brain samples were significantly larger, leading to a greater total aggregated ɑSyn mass. This suggests that even in a region without Lewy body pathology at the studied stage, morphological differences in nanoscopic aggregates exist in disease, indicating that there has already been a disruption of protein homeostasis and, hence, increased aggregation. However, if all neurons showed increased aggregation, then one would expect an increase in aggregate concentration along with an increase in average aggregate size. However, if only a fraction of neurons have disrupted protein homeostasis leading to growth of longer aggregates, this could result in an increase in average length but no change in aggregate concentration. Indeed, fitting the data to our mathematical model showed that the fraction of aggregates from cells with disrupted aggregate removal mechanisms increased from low to undetectable levels in control samples to the majority in samples from PD. This effect was also dependent on the extraction method, with the most pronounced differences in aggregates from soaking and TrX extraction. However, even though the greatest quantity of aggregates was in the homogenized samples, the fraction of aggregates that came from diseased cells showed the smallest difference between PD and control cases for this method.

Both the concentration and morphology of the aggregates differed significantly between the tissue harvesting methods. The least amount of aggregates from both the PD and control brains were harvested by sarkosyl extraction, which also yielded the shortest aggregates in the disease samples. Since sarkosyl partially solubilizes fibrillar aggregates, the lack of Lewy body pathology and in parallel the small amount of insoluble (fibrillar) aggregates in the orbitofrontal cortex at this disease stage explain the low quantity of sarkosyl-soluble aggregates in the samples. Importantly, our kinetic model also showed that the least mechanistic information was contained in sarkosyl-extracted aggregates, as judged by the similarity between diseased, healthy, and mouse samples. This suggests that the size distributions obtained by sarkosyl extraction are dominated by the effects of the harsh extraction conditions that disrupt the morphology of the aggregates, rather than reflecting the size distribution that existed *in situ*. Meanwhile, the soaked and TrX-soluble aggregates showed a higher disease-related signal than the homogenized samples, even though their concentrations were not as high. As shown previously, the diffusible aggregates harvested by gentle soaking may have higher toxic properties,^16^^,^^17^ contributing to the pathogenesis of PD. Similarly, it has been shown that ɑSyn aggregates may grow on the lipid membrane,^20^^,^^22^ and as such, these TrX-harvested aggregates, which would be expected to be membrane-associated, may be more relevant to early PD pathology.

Our previous work imaged aggregates in the soaked fraction in the amygdala, which, unlike the orbitofrontal cortex, shows high levels of pathology and neuronal loss at this stage.^35^ That study also used a different super-resolution method based on an aptamer that imaged beta-sheet-containing aggregates (both beta-amyloid and ɑSyn); thus, the results cannot be directly compared with the current study. Nevertheless, it is noteworthy that in our previous study we have shown smaller aggregates in PD samples compared to controls in the amygdala, suggesting that prior to neuronal loss, larger ɑSyn aggregates form (as seen here in the orbitofrontal cortex), which then possibly leads to the loss of neurons containing these larger aggregates, leaving cells with smaller aggregates behind. This also highlights that in future work it will be informative to compare aggregates in different brain regions at different disease stages using the same imaging method, as PD pathology seems to progress at different rates in different brain regions, with the formation of larger aggregates leading to cell death and related behavioral phenotypes.

Following the characterization of human brain-derived aggregates, we also compared them to the Line 61 mouse model of PD at different ages. Histopathological ɑSyn accumulation starts in Line 61 mice by 1 month of age.^36^ As demonstrated by Rabl et al.,^37^ while the total (monomeric and aggregated) TrX-soluble human ɑSyn levels increase until 6 months of age in the hippocampus, they decrease in the striatum. However, the TrX-insoluble fragments do not change significantly in either region.^37^ By different methods, our results show that the number of aggregates does not change over time, yet their size increases with age, leading to greater total ɑSyn mass in the brains of older mice. As such, the constant presence and slow growth of ɑSyn aggregates may eventually lead to PD-like pathology. However, this age-dependent increase in aggregate size was not detectable with all extraction methods: while the aggregates in the homogenized and TrX-soluble fractions grew larger by 12 months of age, the aggregates in the soaked samples got smaller. This harvesting method-dependent difference in aggregate morphology demonstrates the presence of aggregates accessible by different methods in brain tissue and highlights the importance of studying the relationship between the harvesting method and the aggregates collected.

Compared to the human brain samples, the Line 61 mice had a significantly higher (∼10-fold) aggregate load, probably due to the overexpression of the *SNCA* gene. We have also found that ɑSyn aggregates in the human brain are shorter, with larger total areas, and are rounder compared to those in the Line 61 mouse brain. While the average length of human aggregates was under 100 nm, agreeing with previous findings from our group,^16^ aggregates in the mouse brain—especially those in the homogenized and TrX-soluble fragments—were ∼150 nm in length. The presence of longer aggregates in the mice may also be due to the overexpression of ɑSyn, meaning that aggregate removal processes are overwhelmed in a larger number of cells, resulting in more aggregates than in humans. Humans have less rapid aggregation, and hence removal processes are more effective, so that fewer larger fibrillar aggregates form. Overall, the proportion of aggregates in the different fractions were remarkably similar between humans and the Line 61, suggesting that the aggregate properties that determine the relative amounts in each fraction are similar in both the mouse model and humans. Nevertheless, the morphological differences between the human PD samples and the mouse model are concerning. Since small aggregates can interact with different cellular receptors and other proteins, giving rise to various disease mechanisms, differences in the morphology of these aggregates may reduce the validity of this model. Indeed, the lack of dopaminergic neuronal loss and the heavier pathology seen in the hippocampus may be at least partly due to these differences in small aggregate characteristics, which should be considered when working with this mouse model.

In conclusion, our results show that two populations of ɑSyn aggregates, from healthy and diseased cells, exist in both control and PD brains. This is because neurons in both control and PD brains form and remove nanoscopic aggregates, but the clearance of these aggregates is reduced in diseased cells, which are more abundant in PD., This reduction in relative removal leads to further growth of these aggregates and increased total aggregated ɑSyn mass. This may eventually lead to the formation of Lewy bodies in PD brains, promoting cell death. As such, simply quantifying these aggregates is not sufficient to gain insights into PD mechanisms, and advanced single-molecule imaging methods are required to characterize these nanoscopic aggregates individually, which can then be analyzed using mathematical models to understand the pathogenesis of PD.

### Limitations of the study

While the methods and results presented here demonstrate the utility of single-molecule detection and data modeling for understanding the pathophysiology of PD, this work has a number of limitations. While allowing the characterization of nanoscopic aggregates under the diffraction-limit of light, DNA-PAINT microscopy still has a resolution limit of 20–30 nm, and thus any aggregate smaller than that cannot be resolved. Moreover, factors such as the postmortem interval may have an impact on tissue quality and aggregate morphology in the human brain samples. Lastly, only female Line 61 mice were used in this work, making it impossible to study sex differences. This decision was based on the X chromosome-linked expression of *SNCA* in this model, as stochastic inactivation of the X chromosome may lead to differences in expression between neurons and confound the results. Nevertheless, future work should include sex differences using other synucleinopathy models.

## Resource availability

### Lead contact

Requests for further information and resources should be directed to David Klenerman, Yusuf Hamied Department of Chemistry, University of Cambridge (dk10012@cam.ac.uk).

### Materials availability

This study did not generate new unique reagents.

### Data and code availability

• Data reported in this paper will be shared by the lead contact upon request.
• This paper does not report original code.
• Any additional information required to analyze the data reported in this paper is available from the lead contact upon request.

## Acknowledgments

We would like to thank Rohan T. Ranasinghe for his help during data collection. This work was supported by the UK Dementia Research Institute (which receives its funding from UK DRI Ltd), the UK Medical Research Council, Alzheimer’s Society and Alzheimer’s Research UK (ARUK-PG2020A-009, ARUK-PG2020A-009), the Royal Society, and a grant from Eisai. The views expressed are those of the authors and not necessarily those of the NHS, the NIHR, or the Department of Health.

## Author contributions

E.F. Conception and design, data collection and statistical analysis, manuscript writing. J.S.H.D. Conception and design, data collection and image analysis, manuscript editing. S.N. Conception and design, data collection, manuscript editing. G.M. and M.W.C. Model building and data fitting. M.B., J.Y.L.L., Z.X., Y.W., and B.P. Data collection. Y.I. Conception and design. A.Q. Human brain sample preparation and characterization. J.S. Conception and design, manuscript editing. D.K. Conception and design, manuscript editing, overall supervision of the project.

## Declaration of interests

The authors declare no competing interests.

## STAR★Methods

### Key resources table

**Table:** 

REAGENT or RESOURCE	SOURCE	IDENTIFIER
**Antibodies**		

Mouse Monoclonal anti-alpha Synuclein	Thermo Fisher Scientific	Cat#MA1-90346; RRID: AB_1954821

**Biological samples**		

Healthy human postmortem orbitofrontal cortex brain tissue	Cambridge Brain Bank (Cambridge University Hospitals), Cambridge, United Kingdom	N/A
Parkinson’s disease human postmortem orbitofrontal cortex brain tissue	Cambridge Brain Bank (Cambridge University Hospitals), Cambridge, United Kingdom	N/A

**Chemicals, peptides, and recombinant proteins**		

Artificial CSF	bio-techne TORIS	Cat#3525
Halt™ protease and phosphatase inhibitor single-use cocktail	Thermo Fischer Scientific	Cat#78442
Triton X-100	Fischer Scientific	Cat#11488696
Sarkosyl	Sigma-Aldrich	Cat#61747-100ML
SiMoA® Homebrew carboxylated beads	Quanterix	Cat#104006

**Critical commercial assays**		

Pierce™ BCA Protein Assay Kit	Thermo Fischer Scientific	Cat#23227

**Experimental models: Organisms/strains**		

Mouse: Line 61	Scantox Neuro	RRID:IMSR_JAX:038796

**Software and algorithms**		

The R Project Statistical Computing version 4.2.2 (2022-10-31)—" Innocent and Trusting"	−	https://www.r-project.org/
GraphPad Prism 7.0a	−	https://www.graphpad.com/
Inkscape	−	https://apps.microsoft.com/detail/9pd9bhglfc7h?hl=en-GB&gl=GB
Aggregate Characterisation Tool (ACT)	Xia et al.^38^	https://github.com/zengjiexia/ACT
ASAP	Danial et al.^39^	https://github.com/jdanial/ASAP

### Experimental model and study participant details

3 PD, and 3 age- and sex-matched (two female, one male) postmortem human orbitofrontal cortex (Brodmann areas 10–11) samples were used alongside brain samples from Line 61 mice. The human disease brain samples were selected from Lewy body Braak stages 4/5.^40^ Due to the extensive dopaminergic input to this region from the midbrain, the orbitofrontal cortex is involved in the decision making and reward processing deficits seen in PD.^32^^,^^33^ Brain samples were snap frozen and stored frozen at −80°C until processing. Human postmortem brain tissue was acquired from the Cambridge Brain Bank (Cambridge University Hospitals). The Cambridge Brain Bank is supported by the NIHR Cambridge Biomedical Research Center (NIHR203312). We gratefully acknowledge the participation of all our patient and control volunteers.

The Line 61 model was first developed and described by Rockenstein et al.*,*^41^ as a X chromosome-linked transgenic model, expressing human *SNCA* gene under the murine thymocyte differentiation antigen 1 (Thy1) promoter in C57BL/6xDBA/2 mice.^42^ Unlike many mouse models over-expressing disease-associated proteins at un-physiologically high levels, the Line 61 mice express full-length human ɑSyn at biologically-relevant levels throughout the brain; histopathological and Western blot analysis showed a 1.5- to 3.4-fold rise in expression,^36^ which is comparable to individuals with familial PD due to *SNCA* gene triplication.^43^ While ɑSyn accumulation starts around 1 month of age, no significant dopaminergic or motor neuron loss has been reported, even at advanced ages.^36^^,^^41^ On the other hand, hippocampal neuronal loss -especially in the CA3, is apparent by 3 months of age and deficits in dopamine release and striatal pathology, along with increased gliosis (both astrocytes and microglia), and mitochondrial dysfunction are observed by 6-month of age.^44^^,^^45^^,^^46^^,^^47^ Motor and non-motor behavioral dysfunction has also been reported in this model, starting as early as 1-month and progressing with age.^36^^,^^37^

Due to the appearance of ɑSyn accumulation by 1-month and the pathophysiological symptoms by 6-month of age, and the age-related symptom progression, 1.5-, 6-, 9-, and 12-month-old mouse brains were used in this study, acquired from QPS (now Scantox Neuro). Brains were harvested from a total of 24 male Line 61 (6 mice per group at 1.5-, 6-, 9- and 12-month of age), following euthanasia with sodium phenobarbital (200 mg/kg) and perfusion with phosphate buffered saline (PBS 10%). 12 of the brains were used for the gentle soaking extraction, while the remaining 12 were powdered (homogenised) and serial extractions with artificial cerebrospinal fluid (aCSF), TrX, and sarkosyl were performed (see Graphical Abstract). While the lack of female mice is a limitation of this work, this decision was based on the X chromosome-linked expression of *SNCA* in this model, as the stochastic inactivation of the X chromosome may lead to differences of expression between neurons and confound the results.

### Method details

#### Human tissue collection and sample preparation

Postmortem orbitofrontal cortex samples were first dissected into 300–350 mg pieces (Table 1). For the gentle soaking protocol, tissue samples were first cut into small pieces with a scalpel and transferred to 1.5 mL protein low-binding Eppendorf tubes (Eppendorf, Cat. 0030108116). 600 μL of aCSF buffer (bio-techne TORIS, Cat. 3525) with Halt protease and phosphatase inhibitor single-use cocktail (100x; Thermo Fischer Scientific, Cat. 78442) was added to each sample and incubated for 45-min at 4°C on a HulaMixer™ (Thermo Fischer Scientific, Cat. 15920D). Then the samples were centrifuged at 17,000 G for 120-min at 4°C, the supernatant was collected and stored in a −80°C freezer until analysis. Subsequently, the pellet was resuspended with 500 μL aCSF with protease/phosphatase inhibitors and 1 mm zirconium beads (Scientific Labs, Cat. SLS1414) were added. Then the samples were processed on an electronic tissue homogeniser (VelociRuptor V2 Microtube Homogeniser, Scientific Labs, Cat. SLS1401), at 5 meters/s for 2 cycles of 15 s, with a 10 s gap in between, followed by centrifugation at 17,000 G for 120-min at 4°C. The supernatant was collected and stored in a −80°C freezer until analysis as the “homogenized” sample. Meanwhile, the centrifugation pellet was resuspended with 500 μL aCSF containing inhibitors as described above, as well as 1% (by volume) TrX (Fischer Scientific, Cat. 11488696) and incubated for 45-min at 4°C on a HulaMixer™. Then the samples were centrifuged at 17,000 G for 120-min at 4°C, the supernatant (TrX soluble sample) was collected and stored in a −80°C freezer until analysis. Then the centrifugation pellet was once again resuspended with 500 μL aCSF containing inhibitors as described above, as well as 1% (by volume) sarkosyl (Sigma-Aldrich, Cat. 61747-100ML) and the same extraction described above was performed to acquire the sarkosyl soluble sample (see Graphical Abstract).

#### Mouse sample preparation

For the gentle soaking protocol, mouse cerebral tissue was first cut down into small pieces with a scalpel and transferred to 1.5 mL lo-binding Eppendorf tubes. 600 μL of aCSF buffer with Halt protease and phosphatase inhibitor single-use cocktail was added to each sample and incubated for 45-min at 4°C on a HulaMixer™. Then the samples were centrifuged at 17,000 G for 120-min at 4°C, the supernatant was collected and stored at −80°C until analysis.

For the serial extractions, the intact mouse cerebrum was first frozen with liquid nitrogen and grounded into powder using a mortar and pestle. Then the powder was transferred into a 1.5 mL lo-binding Eppendorf tube and the initial aCSF extraction was performed as described above. The supernatant (homogenized/powder sample) was collected and stored at −80°C until analysis. Subsequently, the pellets were further extracted with TrX and sarkosyl, in the same way as described for the human samples.

For the serial extractions, the intact mouse cerebrum was first frozen with liquid nitrogen and grounded into powder using a mortar and pestle. Then the powder was transferred into a 1.5 mL protein low-binding Eppendorf tube and the initial aCSF extraction was performed as described above. The supernatant (homogenised/powder sample) was collected and stored at −80°C until analysis. Meanwhile, the centrifugation pellet was resuspended with 500 μL aCSF containing inhibitors as described above, as well as 1% (by volume) TrX and incubated for 45-min at 4°C on a HulaMixer™. Then the samples were centrifuged at 17,000 G for 120-min at 4°C, the supernatant (TrX soluble sample) was collected and stored in a −80°C freezer until analysis. Then the centrifugation pellet was once again resuspended with 500 μL aCSF containing inhibitors as described above, as well as 1% (by volume) sarkosyl and the same extraction described above was performed to acquire the sarkosyl soluble sample.

#### BCA assay

Total protein concentration in the samples processed by different methods was determined using a bicinchoninic acid (BCA) assay (Pierce BCA Protein Assay Kit, Thermo Fischer Scientific, Cat. 23227). In brief, samples were diluted in PBS (1:5 by volume) and 25 μL diluted sample was loaded in duplicates into a 96-well plate. Bovine serum albumin (BSA) assay standards were also diluted in PBS at instructed amounts by the kit and loaded alongside the samples, also at 25 μL. Assay working reagent was prepared by mixing 50 parts of BCA Reagent A with 1 part of BCA Reagent B (50:1, Reagent A:B), provided with the kit and added at 200 μL to each sample. The plate was gently shaken for 30 s, covered, and incubated at room temperature for 30 min. Then the absorbance was measured at 562 nm on a plate reader.

#### SIMOA assay

ɑSyn aggregates in the mouse and human brain samples were quantified using an ɑSyn aggregate-specific single-molecule array (SIMOA) we have developed.^26^ SiMoA Homebrew carboxylated beads (Quanterix; Cat.104006) were first functionalised with monoclonal 4B12 antibody (Thermo Fisher Scientific; Cat. MA1-90346) as described in the Quanterix Homebrew instruction manual. In brief, antibodies were buffer-exchanged with the provided bead conjugation buffer and 1 mL of the paramagnetic carboxylated beads (4.2 × 108 beads) were washed with the provided bead wash buffer followed by the bead conjugation buffer with the. 9 μL of EDC solution was added to the washed beads and the reaction mixture was incubated on a mixer for 30 min at 4°C. After washing the activated beads once with cold 25 mM MES, the buffer-changed antibodies (0.2 mg/mL, 300 μL) were added. The reaction mixture was incubated on a mixer at 4°C for two hours. The conjugated beads were then washed with the provided bead wash buffer and blocked with the blocking buffer at room temperature for 40 min.

The beads coated with the 4B12 antibody were first washed three times with the provided bead diluent in the kit. Meanwhile, to each well on a conical bottom microplate (Quanterix), conditioned media samples were diluted with the lysate sample diluent (2:98) to give 100 μL solution at the desired concentration. Then, 25 μL of the washed beads were introduced to each well to give the final-bead concentration as 2 × 10^7^ beads/mL. The samples and the beads were incubated on the plate shaker at 30°C at 800 revolutions per minute (rpm) for 30 min. The plate was then washed by the SIMOA washer with the provided buffers. Then, the biotinylated detector antibody 4B12 was diluted to 0.1 μg/mL with the provided Homebrew detector diluent and 100 μL of the diluted detectors were introduced to each well. The mixture was then incubated on the plate shaker at 30°C with 800 rpm for 10 min, followed by another wash. Meanwhile, the provided streptavidin-β-galactosidase (SBG) concentrate was diluted to 50 pM with SBG diluent. 100 μL of the diluted SBG was added to each well and the mixture was incubated on the plate shaker at 30°C at 800 rpm for 10 min. Finally, the plate was washed again and loaded onto the SR-X Biomarker Detection System (Quanterix) together with the SIMOA disc, tips, and an RGP bottle.

#### DNA-PAINT with SiMPull imaging

ɑSyn aggregates in the mouse and human brain samples were characterised in terms of their size and shape using DNA point accumulation in nanoscale topography (DNA-PAINT) microscopy combined with single-molecule pulldown (SiMPull^27^; see Graphical Abstract). SiMPull coverslips were prepared as described previously^16^^,^^27^ and kept in vacuumed containers at −20°C until used. At the beginning of each experiment, one vacuum box containing the coverslips was taken out of the freezer and placed inside a fume-hood to allow equalisation of the temperature. The coverslip was first coated with neutravidin (0.2 mg/mL) diluted in TBST(v/v) (0.05% Tween 20 Cat. P1379-25ML diluted in tris-buffered saline (TBS)) for 10-min. After removing the neutravidin solution, each well was washed with TBST (two cycles) and then with TBS with 1% Tween (one cycle)-hereon described as washing. Biotinylated 4B12 antibody was diluted to 10 nM in TBS containing 0.1 mg/mL bovine serum albumin (BSA; Thermo Scientific; Cat. 10829410) and incubated for 15 min. Then the wells were washed, and samples were incubated overnight at 4°C. While the cut, powder, and sarkosyl extracted samples were tested neat, the TrX extracted sample was diluted in TBS 50:50 by volume. In order to minimise non-specific binding, a blocking step was performed after samples incubation, with solution containing 3 mg/mL BSA in TBS, incubated for 30 min. Then the DNA-labelled 4B12 antibody diluted to 2 nM in TBS containing 0.1 mg/mL bovine serum albumin was incubated for 30 min (labeling protocol explained by Fertan et al.^27^). After the washing steps, TetraSpeck microspheres (1:7,000 in TBS, 10 μL, Thermo Scientific; Cat. T7279) were introduced to each well for 10 min. The TetraSpeck solution was then removed, followed by a single wash with TBST, and a second PDMS gasket (Merck, GBL-103250-10EA) was stacked on the coverslip before introducing 4 μL of imaging strand (TGGTGGT-cy3B; atdbio) in TBS. Finally, the coverslip was sealed with another coverslip on top of the second PDMS gasket. The coverslip was imaged using a purpose built total internal reflection fluorescence (TIRF) microscope^48^ using a 568-nm (green) excitation laser for 4,000 frames per field of view (FoV) at an exposure time of 100 ms. As measured previously^48^ using DNA origami structures,^49^ the spatial resolution of this setup is ∼30 nanometres. Aggregates below this length were not included in the analyses.

### Quantification and statistical analysis

#### Data analyses

All BCA and SIMOA assays were run in duplicates, and the data were averaged. DNA-PAINT imaging was performed for 4 FoVs per well and two wells for each sample, from which the data were pooled. Super-resolution images were reconstructed, drift corrected, and analyzed using a software developed by our group.^38^^,^^50^ The R Project Statistical Computing version 4.2.2 (2022-10-31)—“Innocent and Trusting” was used for all statistical analyses, the graphs were generated in GraphPad Prism 7.0a for Mac OS X and Inkscape, and the cartoon figures were created with BioRender.com. Linear mixed effects models, ANOVAs with Type 2 sums of squares, and Welch Two Sample t-tests were used to determine differences between the groups and corresponding 95% confidence intervals (CI) were reported. Mean ± standard error of mean (SEM) are presented on the graphs. The datasets generated and analyzed during the current study are available from the corresponding author on reasonable request.

#### Distribution fitting

For the detailed derivation of our models see Cotton et al.^25^ Briefly, we assume that at aggregated sizes well above the nucleus size, the number of aggregates at each size is determined by the rate at which they grow compared to the rate at which they are removed, through any process. This predicts a geometrically decaying size distribution, i.e., a straight line when the histograms of aggregate population versus size are plotted on a logarithmic plot (Figures 6A and 6B). We expect this to describe the situation within an individual cell. To analyze the aggregate size distributions from a brain sample, we need to consider the fact that it will contain aggregates from many different cells, some of which may be significantly more affected by disease/contain significantly more aggregates than others. At the very least, we expect there to be two cell states a healthy one and a disease associated one, in which the relative rates of aggregate production and removal, and thus the length distributions, differ. There is evidence that this binary state is indeed a good description.^25^^,^^39^ We therefore fit the data to a model that allows 2 different cell states: a healthy one in which aggregate production is slower compared to removal, determined by the parameter *α*_*H*_, and a diseased cell state in which aggregate production is faster compared to removal, determined by the parameter *α*_*D*_. The free parameters are the relative rates of aggregate removal in each cell state and the fraction of aggregates from the diseased state. To determine these parameters from data, we convert the length in nm to a length in terms of the number of proteins by multiplying by 4 (assuming a periodicity of 2 monomers per nm and a double stranded fibril). We then use Bayesian inference, where the likelihood function of an aggregate to be x monomers long is given by

$$p\left(x\right)=\beta \frac{\alpha_{D}^{x}\left(1-\alpha_{D}\right)}{\alpha_{D}^{nmin}-\alpha_{D}^{nmax+1}}+\left(1-\beta \right)\frac{\alpha_{H}^{x}\left(1-\alpha_{H}\right)}{\alpha_{H}^{nmin}-\alpha_{H}^{nmax+1}}$$

where *x* is the length, *nmin* the minimum aggregate size considered and *nmax* the maximum aggregate size considered. The parameter *α* is related to the rates of growth, *g*, and removal, *r*, by $\alpha ={\left(\frac{r}{g}+1\right)}^{-1}$

While we use this quantity to fit the aggregate length distribution, in the main text we refer to the relative removal rate *r/g* as that is a more easily interpreted parameter. The minimum size is set to exclude the smallest aggregates where nucleation processes are expected to be important and the assumptions for our length distribution model are thus no longer valid (for details see Cotton et al.^25^). We chose a minimum cutoff, *nmin,* of 500 monomers and a maximum cutoff (*nmax*) of 3000, which prevents outlier effects from single very large aggregates.

To determine *α*_*H*_, *α*_*D*_ and the fraction of aggregates from diseased cells from experimental data, we perform Bayesian inference, which allows us to directly use each measured length to determine the posterior probability rather than requiring binning and the calculation of histograms. The histograms and “best fit” lines shown in the figures are simply for visualisation.

We assume that, within each extraction method, the healthy and diseased cell states are the same across individuals and stages of the disease (*α*_*H*_ and *α*_*D*_ are global parameters) but allow the fraction of aggregates from diseased cells to differ between individuals. Priors on the parameters are assumed to be constant between 0 and 1. Posteriors are shown in Figure S2. Even with these restrictive conditions on the parameters, the fits of the experimental data are remarkably good (see Figure 4B), showing the distribution from the mean parameters superimposed on a histogram of the data.

## Supplemental information

Document S1. Figures S1−S6

Data S1. Supplemental text containing the results of the statistical tests To improve the clarity and readability of the main text, the results are presented without statistical tests. These statistical results are provided separately in Data S1.

Document S2. Article plus supplemental information

## References

[1] Simon D.K., Tanner C.M., Brundin P. (2020). Parkinson Disease Epidemiology, Pathology, Genetics and Pathophysiology. Clin. Geriatr. Med..

[2] Xia R., Mao Z.H. (2012). Progression of motor symptoms in Parkinson’s disease. Neurosci. Bull..

[3] Smith K.M., Caplan D.N. (2018). Communication impairment in Parkinson’s disease: Impact of motor and cognitive symptoms on speech and language. Brain Lang..

[4] Cosgrove J., Alty J.E., Jamieson S. (2015). Cognitive impairment in Parkinson’s disease. Postgrad. Med. J..

[5] Zhao N., Yang Y., Zhang L., Zhang Q., Balbuena L., Ungvari G.S., Zang Y.F., Xiang Y.T. (2021). Quality of life in Parkinson’s disease: A systematic review and meta-analysis of comparative studies. CNS Neurosci. Ther..

[6] Pinter B., Diem-Zangerl A., Wenning G.K., Scherfler C., Oberaigner W., Seppi K., Poewe W. (2015). Mortality in Parkinson’s disease: a 38-year follow-up study. Mov. Disord..

[7] Parent M., Parent A. (2010). Substantia nigra and Parkinson’s disease: a brief history of their long and intimate relationship. Can. J. Neurol. Sci..

[8] Spillantini M.G., Schmidt M.L., Lee V.M., Trojanowski J.Q., Jakes R., Goedert M. (1997). Alpha-synuclein in Lewy bodies. Nature.

[9] Stefanis L. (2012). α-Synuclein in Parkinson’s Disease. Cold Spring Harb. Perspect. Med..

[10] Diao J., Burré J., Vivona S., Cipriano D.J., Sharma M., Kyoung M., Südhof T.C., Brunger A.T. (2013). Native α-synuclein induces clustering of synaptic-vesicle mimics via binding to phospholipids and synaptobrevin-2/VAMP2. eLife.

[11] Gibb W.R., Lees A.J. (1988). The relevance of the Lewy body to the pathogenesis of idiopathic Parkinson’s disease. J. Neurol. Neurosurg. Psychiatry.

[12] Strohäker T., Jung B.C., Liou S.H., Fernandez C.O., Riedel D., Becker S., Halliday G.M., Bennati M., Kim W.S., Lee S.J. (2019). Structural heterogeneity of α-synuclein fibrils amplified from patient brain extracts. Nat. Commun..

[13] Ingelsson M. (2016). Alpha-Synuclein Oligomers-Neurotoxic Molecules in Parkinson’s Disease and Other Lewy Body Disorders. Front. Neurosci..

[14] Choi B.K., Choi M.G., Kim J.Y., Yang Y., Lai Y., Kweon D.H., Lee N.K., Shin Y.K. (2013). Large α-synuclein oligomers inhibit neuronal SNARE-mediated vesicle docking. Proc. Natl. Acad. Sci. USA.

[15] Yoo G., Yeou S., Son J.B., Shin Y.-K., Lee N.K. (2021). Cooperative inhibition of SNARE-mediated vesicle fusion by α-synuclein monomers and oligomers. Sci. Rep..

[16] Emin D., Zhang Y.P., Lobanova E., Miller A., Li X., Xia Z., Dakin H., Sideris D.I., Lam J.Y.L., Ranasinghe R.T. (2022). Small soluble α-synuclein aggregates are the toxic species in Parkinson’s disease. Nat. Commun..

[17] Hong W., Wang Z., Liu W., O’Malley T.T., Jin M., Willem M., Haass C., Frosch M.P., Walsh D.M. (2018). Diffusible, highly bioactive oligomers represent a critical minority of soluble Aβ in Alzheimer’s disease brain. Acta Neuropathol..

[18] Fertan E., Hung C., Danial J.S.H., Lam J.Y.L., Preman P., Albertini G., English E.A., Böken D., Livesey F.J., De Strooper B. (2025). Clearance of beta-amyloid and tau aggregates are size-dependent and altered by an inflammatory challenge. Brain Commun..

[19] Hong W., Liu W., Desousa A.O., Young-Pearse T., Walsh D.M. (2023). Methods for the isolation and analysis of Aβ from postmortem brain. Front. Neurosci..

[20] Šneiderienė G., Czekalska M.A., Xu C.K., Jayaram A.K., Krainer G., Arter W.E., Peter Q.A.E., Castellana-Cruz M., Saar K.L., Levin A. (2024). α-Synuclein Oligomers Displace Monomeric α-Synuclein from Lipid Membranes. ACS Nano.

[21] Tsigelny I.F., Sharikov Y., Wrasidlo W., Gonzalez T., Desplats P.A., Crews L., Spencer B., Masliah E. (2012). Role of α-synuclein penetration into the membrane in the mechanisms of oligomer pore formation. FEBS J..

[22] Dear A.J., Teng X., Ball S.R., Lewin J., Horne R.I., Clow D., Stevenson A., Harper N., Yahya K., Yang X. (2024). Molecular mechanism of α-synuclein aggregation on lipid membranes revealed. Chem. Sci..

[23] Stojkovska I., Mazzulli J.R. (2021). Detection of pathological alpha-synuclein aggregates in human iPSC-derived neurons and tissue. STAR Protoc..

[24] Gram H., Theologidis V., Boesen T., Jensen P.H. (2023). Sarkosyl differentially solubilizes patient-derived alpha-synuclein fibril strains. Front. Mol. Biosci..

[25] Cotton M.W., Venkatesan S., Beckwith J.S., Böken D., Xu C.K., Fertan E., Breiter J.C., Berkowicz L.E., Salazar L.S., Schulze A.V. (2025). Neurodegeneration emerges at a cellular tipping point between aggregate accumulation and removal. bioRxiv.

[26] Böken D., Xia Z., Lam J.Y.L., Fertan E., Wu Y., English E.A., Konc J., Layburn F., Bernardes G.J.L., Zetterberg H. (2024). Ultrasensitive Protein Aggregate Quantification Assays for Neurodegenerative Diseases on the Simoa Platform. Anal. Chem..

[27] Fertan E., Böken D., Murray A., Danial J.S.H., Lam J.Y.L., Wu Y., Goh P.A., Alić I., Cheetham M.R., Lobanova E. (2023). Cerebral organoids with chromosome 21 trisomy secrete Alzheimer’s disease-related soluble aggregates detectable by single-molecule-fluorescence and super-resolution microscopy. Mol Psychiatry.

[28] Böken D., Cox D., Burke M., Lam J.Y.L., Katsinelos T., Danial J.S.H., Fertan E., McEwan W.A., Rowe J.B., Klenerman D. (2024). Single-Molecule Characterization and Super-Resolution Imaging of Alzheimer’s Disease-Relevant Tau Aggregates in Human Samples. Angew. Chem. Int. Ed. Engl..

[29] Jungmann R., Avendaño M.S., Dai M., Woehrstein J.B., Agasti S.S., Feiger Z., Rodal A., Yin P. (2016). Quantitative super-resolution imaging with qPAINT. Nat. Methods.

[30] Bousset L., Pieri L., Ruiz-Arlandis G., Gath J., Jensen P.H., Habenstein B., Madiona K., Olieric V., Böckmann A., Meier B.H., Melki R. (2013). Structural and functional characterization of two alpha-synuclein strains. Nat. Commun..

[31] Dirnberger G., Jahanshahi M. (2013). Executive dysfunction in Parkinson’s disease: a review. J. Neuropsychol..

[32] du Plessis S., Bossert M., Vink M., van den Heuvel L., Bardien S., Emsley R., Buckle C., Seedat S., Carr J. (2018). Reward processing dysfunction in ventral striatum and orbitofrontal cortex in Parkinson’s disease. Parkinsonism Relat. Disord..

[33] Kobayakawa M., Tsuruya N., Kawamura M. (2017). Decision-making performance in Parkinson’s disease correlates with lateral orbitofrontal volume. J. Neurol. Sci..

[34] Tinaz S., Courtney M.G., Stern C.E. (2011). Focal cortical and subcortical atrophy in early Parkinson’s disease. Mov. Disord..

[35] Harding A.J., Stimson E., Henderson J.M., Halliday G.M. (2002). Clinical correlates of selective pathology in the amygdala of patients with Parkinson’s disease. Brain.

[36] Chesselet M.F., Richter F., Zhu C., Magen I., Watson M.B., Subramaniam S.R. (2012). A progressive mouse model of Parkinson’s disease: the Thy1-aSyn (“Line 61”) mice. Neurotherapeutics.

[37] Rabl R., Breitschaedel C., Flunkert S., Duller S., Amschl D., Neddens J., Niederkofler V., Rockenstein E., Masliah E., Roemer H., Hutter-Paier B. (2017). Early start of progressive motor deficits in Line 61 α-synuclein transgenic mice. BMC Neurosci..

[38] Danial J.S.H., Garcia-Saez A.J. (2019). Quantitative analysis of super-resolved structures using ASAP. Nat. Methods.

[39] Thompson T.B., Meisl G., Knowles T.P.J., Goriely A. (2021). The role of clearance mechanisms in the kinetics of pathological protein aggregation involved in neurodegenerative diseases. J. Chem. Phys..

[40] Braak H., Braak E. (1991). Neuropathological stageing of Alzheimer-related changes. Acta Neuropathol..

[41] Rockenstein E., Mallory M., Hashimoto M., Song D., Shults C.W., Lang I., Masliah E. (2002). Differential neuropathological alterations in transgenic mice expressing alpha-synuclein from the platelet-derived growth factor and Thy-1 promoters. J. Neurosci. Res..

[42] Richter F., Stanojlovic M., Käufer C., Gericke B., Feja M. (2023). A Mouse Model to Test Novel Therapeutics for Parkinson’s Disease: an Update on the Thy1-aSyn (“line 61”) Mice. Neurotherapeutics.

[43] Chartier-Harlin M.-C., Kachergus J., Roumier C., Mouroux V., Douay X., Lincoln S., Levecque C., Larvor L., Andrieux J., Hulihan M. (2004). Alpha-synuclein locus duplication as a cause of familial Parkinson’s disease. Lancet.

[44] Wrasidlo W., Tsigelny I.F., Price D.L., Dutta G., Rockenstein E., Schwarz T.C., Ledolter K., Bonhaus D., Paulino A., Eleuteri S. (2016). A de novo compound targeting α-synuclein improves deficits in models of Parkinson’s disease. Brain.

[45] Kim C., Ojo-Amaize E., Spencer B., Rockenstein E., Mante M., Desplats P., Wrasidlo W., Adame A., Nchekwube E., Oyemade O. (2015). Hypoestoxide reduces neuroinflammation and α-synuclein accumulation in a mouse model of Parkinson’s disease. J. Neuroinflamm..

[46] Price D.L., Koike M.A., Khan A., Wrasidlo W., Rockenstein E., Masliah E., Bonhaus D. (2018). The small molecule alpha-synuclein misfolding inhibitor, NPT200-11, produces multiple benefits in an animal model of Parkinson’s disease. Sci. Rep..

[47] Subramaniam S.R., Vergnes L., Franich N.R., Reue K., Chesselet M.-F. (2014). Region specific mitochondrial impairment in mice with widespread overexpression of alpha-synuclein. Neurobiol. Dis..

[48] Danial J.S.H., Lam J.Y.L., Wu Y., Woolley M., Dimou E., Cheetham M.R., Emin D., Klenerman D. (2022). Constructing a cost-efficient, high-throughput and high-quality single-molecule localization microscope for super-resolution imaging. Nat. Protoc..

[49] Schnitzbauer J., Strauss M.T., Schlichthaerle T., Schueder F., Jungmann R. (2017). Super-resolution microscopy with DNA-PAINT. Nat. Protoc..

[50] Xia Z., Wu Y., Lam J.Y.L., Zhang Z., Burke M., Fertan E., Ranasinghe R.T., Hidari E., Danial J.S.H., Klenerman D. (2023). A computational suite for the structural and functional characterization of amyloid aggregates. Cell Rep. Methods.

