# Supplementary material for: Single-molecule detection methods to study alpha-synuclein aggregation in postmortem Parkinson’s disease brains
Source: Cell Rep Methods. 2026 Apr 23;6(6):101418. doi: 10.1016/j.crmeth.2026.101418 (PMC13282648; doi:10.1016/j.crmeth.2026.101418)
Supplement: Document S1. Figures S1−S6 [file mmc1.pdf]

Cell Reports Methods, Volume *6*

Supplemental information

Single-molecule detection methods

to study alpha-synuclein aggregation

in postmortem Parkinson's disease brains

Emre Fertan, John S.H. Danial, Stephen Neame, Jeff Y.L. Lam, Matthew W. Cotton, Melanie Burke, Zengjie Xia, Yunzhao Wu, Ben Powney, Yoichi Imaizumi, Annelies Quaegebeur, Georg Meisl, James Staddon, and David Klenerman

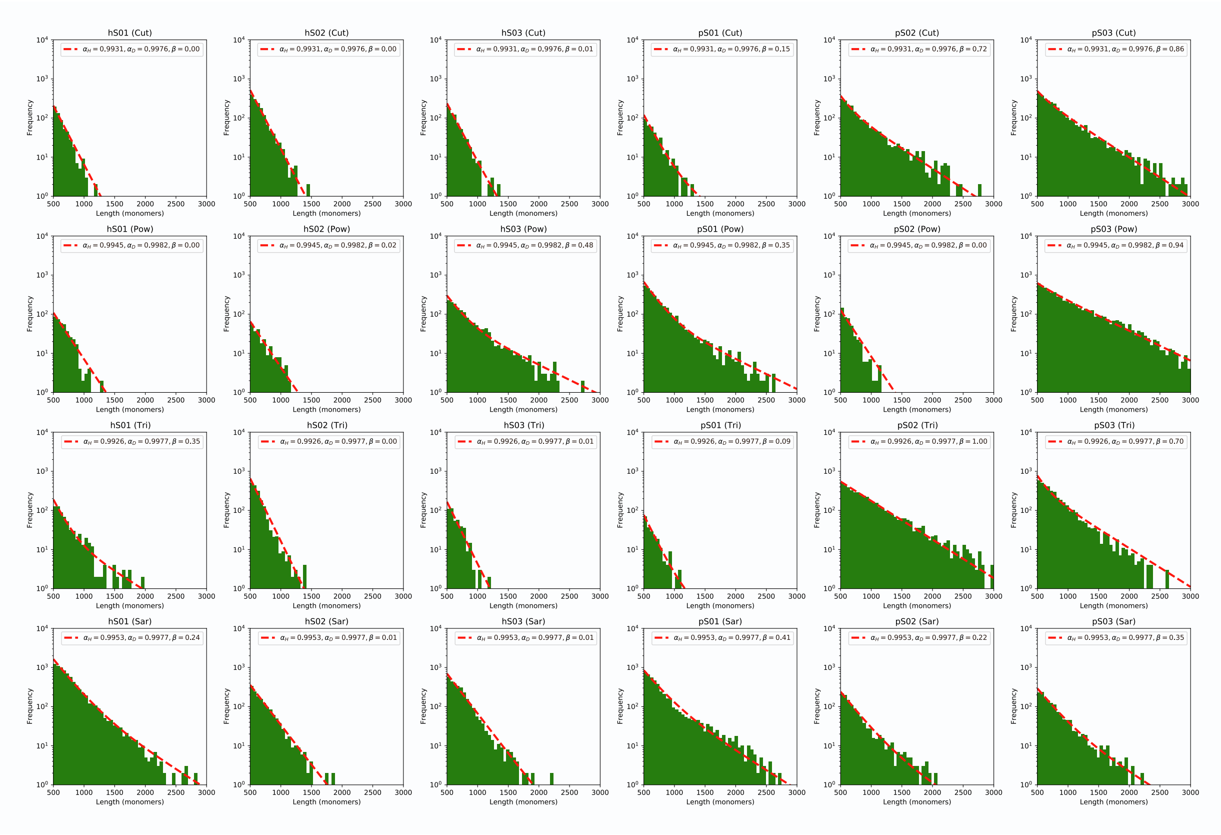

Supplemental Figure 1. Histograms of the measured aggregate length distributions in human samples for different extraction techniques (Related to Figure 4). The red dashed line shows the predicted distribution using the model and the mean parameters determined from the Bayesian inference.

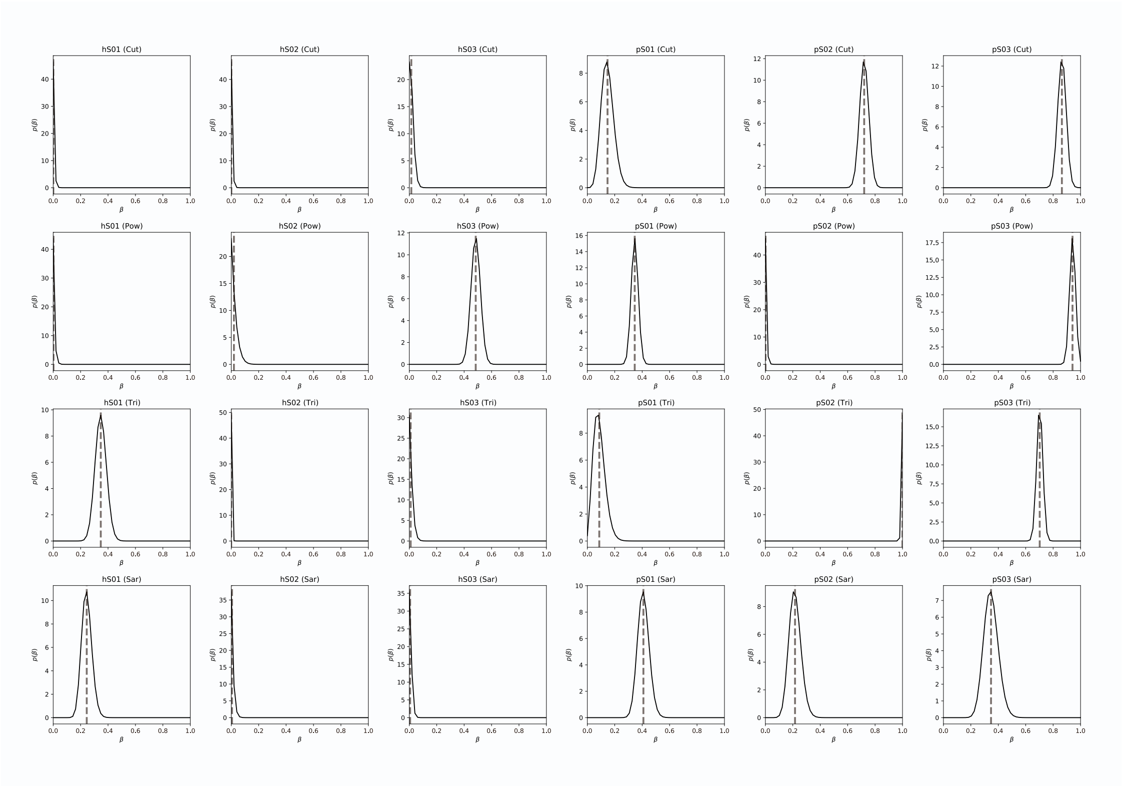

Supplemental Figure 2. Posterior distributions for the fraction of aggregates from diseased cells following the Bayesian inference fitting procedure (Related to Figure 4). The dashed black line shows the mean value for each experiment.

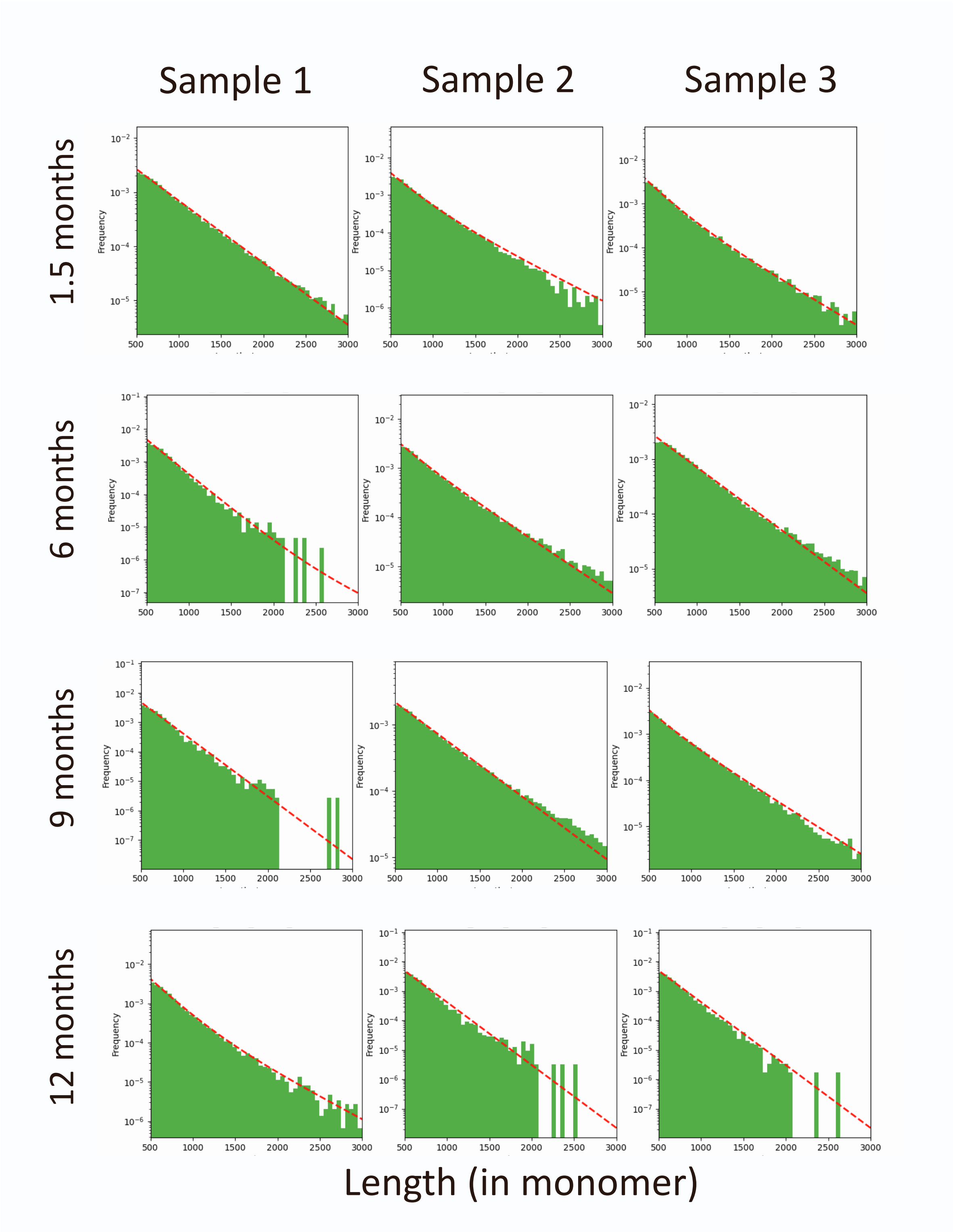

Supplemental Figure 3. Histograms of the measured aggregate length distributions in mouse samples for soaked (cut) samples (Related to Figure 4). The red dashed line shows the predicted distribution using the model and the mean parameters determined from the Bayesian inference.

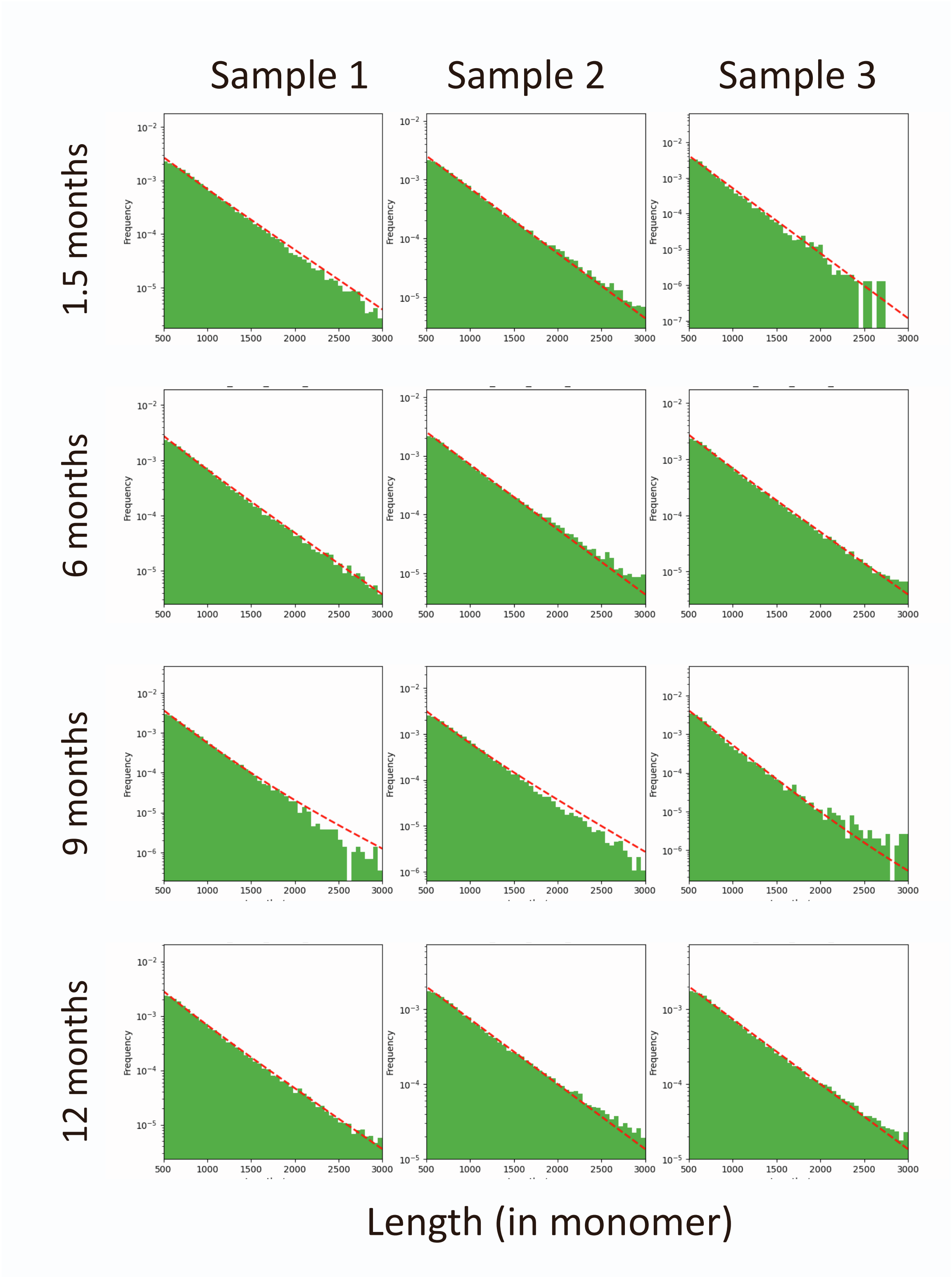

Supplemental Figure 4. Histograms of the measured aggregate length distributions in mouse samples for homogenised (powder) samples (Related to Figure 4). The red dashed line shows the predicted distribution using the model and the mean parameters determined from the Bayesian inference.

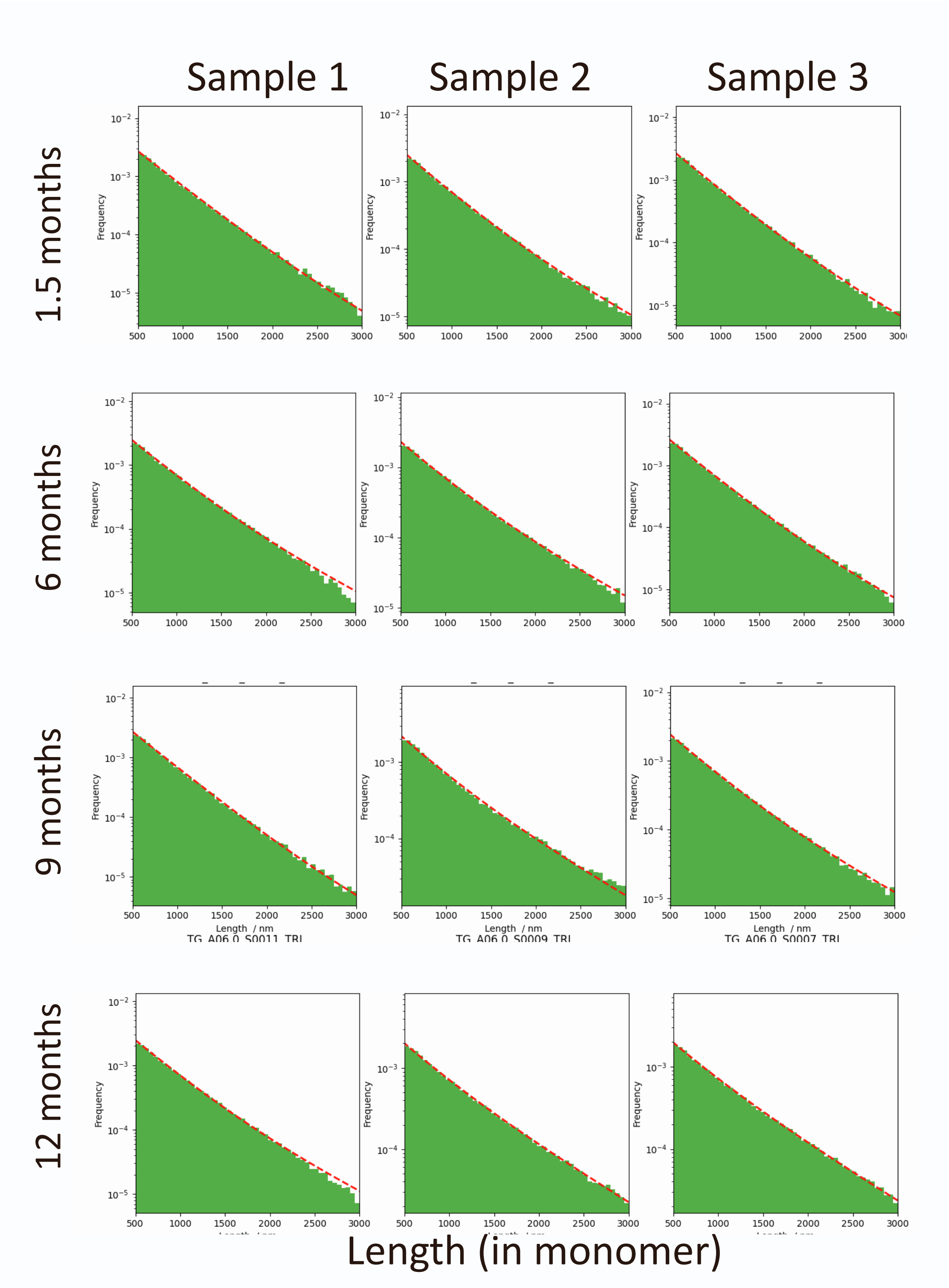

Supplemental Figure 5. Histograms of the measured aggregate length distributions in mouse samples for Triton X-100 extracted samples (Related to Figure 4). The red dashed line shows the predicted distribution using the model and the mean parameters determined from the Bayesian inference.

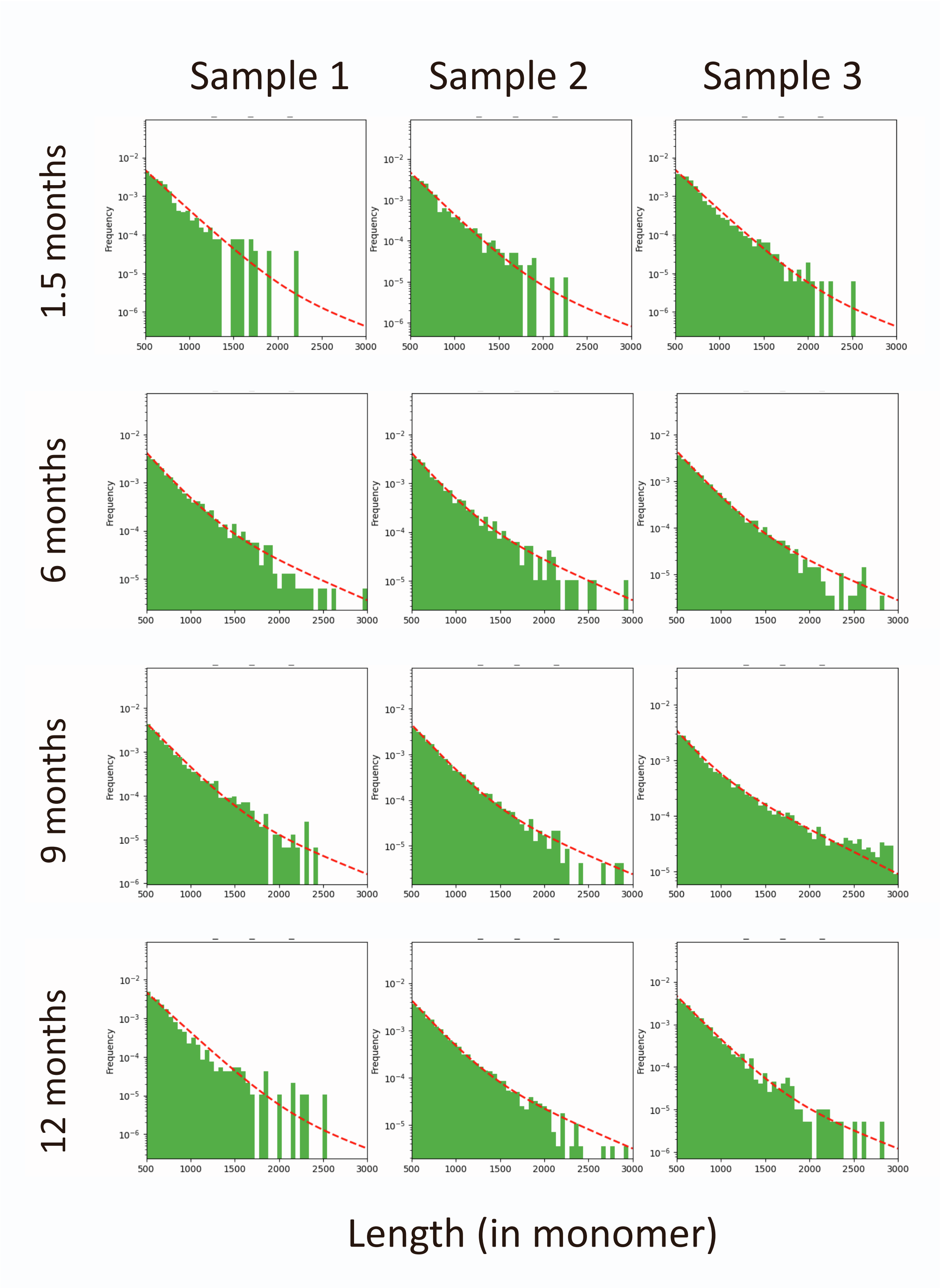

Supplemental Figure 6. Histograms of the measured aggregate length distributions in mouse samples for sarkosyl extracted samples (Related to Figure 4). The red dashed line shows the predicted distribution using the model and the mean parameters determined from the Bayesian inference.
