# Supplementary material for: Single-molecule detection methods to study alpha-synuclein aggregation in postmortem Parkinson’s disease brains
Source: Cell Rep Methods. 2026 Apr 23;6(6):101418. doi: 10.1016/j.crmeth.2026.101418 (PMC13282648; doi:10.1016/j.crmeth.2026.101418)
Supplement: Data S1. Supplemental text containing the results of the statistical tests — To improve the clarity and readability of the main text, the results are presented without statistical tests. These statistical results are provided separately in Data S1. [file mmc2.pdf]

1. αSyn aggregate concentration in PD and control brain: Strikingly, the αSyn aggregate concentrations did not differ between the PD brains and the control samples (**$t_{2.35}$ = 0.31, p = 0.768; Figure 3a**), there was a significant difference between aggregates obtained using the different extraction methods (**$AIC_{Extraction}$ = 22.6, LR = 38.06, p < 0.001; Figures 3a & 4)**. The sarkosyl soluble fragment contained the least number of aggregates for both phenotypes (**CI_{95} = 0.034, 0.479**), followed by the soaked and TrX extracted fractions, and the homogenised fragments contained the highest concentration of αSyn aggregates (**CI_{95} = 0.407, 0.715**).

2. PD brains contain larger aggregates than controls: While the concentration of αSyn aggregates did not differ between the PD and control orbitofrontal cortex samples, morphology of the aggregates differed significantly, as the PD brains contained longer (80 vs 65 nm; **CI_{95} = 13.62, 14.42**) and larger (1360 vs 770 nm^2; **CI_{95} = 579.38, 600.44**) aggregates, with rounder (less fibrillar) shapes (**CI_{95} = 0.011, 0.013**), regardless of extraction method, leading to higher total mass of aggregated αSyn in the PD brains (**$t_{10.66}$ = 2.38, p = 0.037, CI_{95} = 25,972.05, 704,420.84; Figure 3b**).

For the control brains, the longest (**CI_{95}Homogenised = 17.05, 18.76; CI_{95}Sarkosyl = 13.87, 14.93; Figure 5a**), largest (**CI_{95}Homogenised = 553.86, 583.14; CI_{95}Sarkosyl = 255.35, 273.48; Figure 5b**), and roundest (**CI_{95}Homogenised = 0.007, 0.013; CI_{95}Sarkosyl = 0.003, 0.007; Figure 5c**) aggregates were harvested by homogenising and sarkosyl extraction. On the other hand, for the PD samples, the homogenised (**CI_{95} = 12.58, 14.72**) and TrX (**CI_{95} = 9.91, 12.05**) extracted aggregates were longer (**Figure 5d)** and larger (**CI_{95}Homogenised = 676.80, 739.17; CI_{95}TrX = 184.46, 246.81; Figure 5e**), while the shortest (**CI_{95} = 15.54, 17.50**) and smallest (**CI_{95} = 290.36, 347.33**) aggregates were in the sarkosyl extracted samples. Unlike the control brains, the larger aggregates were more fibrillar in the PD brain, as the aggregates with the highest eccentricity were found in the homogenised fraction (**CI_{95} = 0.011, 0.017**), followed by the TrX extraction (**CI_{95} = 0.001, 0.006**), and the roundest aggregates were in the sarkosyl soluble fraction (**CI_{95} = 0.016, 0.021; Figure 5f**).

3. αSyn aggregate concentration in Line 61 mouse brain: While the αSyn aggregate concentration did not differ between the ages (**AIC_{Age} = 133.01, LR = 7.25, p = 0.064; Figure 7a-d**), concentration of aggregates harvested by homogenising, TrX, and sarkosyl extractions

differed significantly (**$AIC_{Extraction}$ = 195.20, LR = 67.44, $p < 0.001$**). The highest concentration of αSyn aggregates was found in the homogenised samples, followed by the TrX soluble extract (**CI_{95} = -6.65, -4.27**), and least amount of aggregates were in the sarkosyl soluble fragment (**CI_{95} = -7.64, -5.25**; **Figures 7e-h**). Meanwhile, the soaked sections, which were prepared from a different brain sample contained a smaller amount of aggregates, compared to the other extraction methods.

<u>4. αSyn aggregate morphology in Line 61 mouse brain:</u> For the soaked samples, aggregate length was 100 nm at 1.5-months of age and increased to 114 and 123 nm by 6- (**CI_{95} = 13.18, 13.88**) and 9-months (**CI_{95} = 22.30, 22.97**) of age, but decreased to an average of 74 nm at 12-months of age (**CI_{95} = -26.18, -26.99**), suggesting a change in the morphology of soaked aggregates as the Line 61 mice age (**Figures 8a&e**). Aggregate area from the soaked samples followed a similar trend to length, with an increase at 9-months of age (**CI_{95} = 200.47, 208.66**), followed by a decrease at 12-months (**CI_{95} = -235.32, -225.60; Figures 8a&i**). Meanwhile, aggregate eccentricity, which is a measure of how fibrillar the aggregate is, did not change with age and had a value close to 1, indicating the presence of more fibrillar aggregates in all samples (**Figures 8a&m**). Within the serially extracted samples, the homogenised (**CI_{95} = 68.92, 69.65**) and TrX soluble (**CI_{95} = 82.34, 83.05**) aggregates had an average length of 128 and 142 nm respectively, and thus were longer than the sarkosyl soluble aggregates, which had an average length of 59 nm. However, while the TrX soluble aggregates steadily grew longer as the mice aged (**CI_{95}6-months = 8.06, 8.78; CI_{95}9-months = 10.38, 11.11; CI_{95}12-months = 27.50, 28.22**), the homogenised aggregates varied in length but showed no clear trend with age (**CI_{95}6-months = 13.85, 14.58; CI_{95}9-months = -15.29, -14.50; CI_{95}12-months = 32.37, 33.09**). In contrast, while the sarkosyl soluble aggregates grew longer after 1.5-months of age (**CI_{95}6-months = 13.51, 14.48**), they did not show any further significant age-related changes in length (**CI_{95}9-months = -0.32, 0.58; CI_{95}12-months = -3.72, -2.77; Figures 8b-d&f-h**). The area of the TrX soluble aggregates grew larger with age (**CI_{95}6-months = 82.03, 90.17; CI_{95}9-months = 141.65, 149.97; CI_{95}12-months = 279.80, 287.94**), while homogenised (**CI_{95}6-months = 19.50, 28.01; CI_{95}9-months = -197.09, -187.94; CI_{95}12-months = 331.62, 339.96**) and sarkosyl soluble (**CI_{95}6-months = 274.09, 294.29; CI_{95}9-months = -108.68, -89.90; CI_{95}12-months = -61.92, -42.22**) aggregates had a fluctuating pattern (**Figures 8b-d&j-l**). On the other hand, eccentricity of the serially extracted samples differed by extraction method (**AIC = 23494277, F = 27151, $p < 0.001$**). While the homogenised (**CI_{95} = 0.063, 0.064**) and TrX

soluble (**CI_{95} = 0.06, 0.06**) aggregates were more fibrillar, the sarkosyl soluble aggregates were more circular (**Figures 8b-d&n-p**).
